# Structure of an innexin gap junction channel and cryo-EM sample preparation

**DOI:** 10.1093/jmicro/dfx035

**Published:** 2017-09-21

**Authors:** Atsunori Oshima

**Affiliations:** 1 Cellular and Structural Physiology Institute (CeSPI), Nagoya University, Furo-cho, Chikusa-ku, Nagoya 464-8601, Japan; 2 Department of Basic Medicinal Sciences, Graduate School of Pharmaceutical Sciences, Nagoya University, Furo-cho, Chikusa-ku, Nagoya 464-8601, Japan

**Keywords:** innexin, connexin, gap junction channel, cryo-electron microscopy, sample preparation, single particle analysis

## Abstract

Gap junction channels are essential for mediating intercellular communication in most multicellular organisms. Two gene families encode gap junction channels, innexin and connexin. Although the sequence similarity between these two families based on bioinformatics is not conclusively determined, the gap junction channels encoded by these two gene families are structurally and functionally analogous. We recently reported an atomic structure of an invertebrate innexin gap junction channel using single-particle cryo-electron microscopy. Our findings revealed that connexin and innexin families share several structural properties with regard to their monomeric and oligomeric structures, while simultaneously suggesting a diversity of gap junction channels in nature. This review summarizes cutting-edge progress toward determining an innexin gap junction channel structure, as well as essential tips for preparing cryo-electron microscopy samples for high-resolution structural analysis of an innexin gap junction channel.

## Teaser

An atomic structure of the Caenorhabditis elegans innexin-6 gap junction channel has recently been determined using single-particle cryo-electron microscopy. This review summarizes progress toward determining the innexin-6 gap junction channel structure and essential tips for preparing cryo-electron microscopy samples for high-resolution structural analysis of an innexin gap junction channel.

## Introduction

Gap junction channels enable direct intercellular transfer of small permeants, whereby the adjacent cells are coupled electrically and chemically. Two gene families form gap junction channels, connexin and innexin. A connexin gap junction channel comprises 12 subunits in which hexameric hemichannels are opposed to each other to form a full gap junction channel, and the connexin family contains more than 20 isoforms. The completed genome project of model organisms such as *Drosophila melanogaster* and *Caenorhabditis elegans* revealed that invertebrates have no connexin homologue proteins [1,2]. Following a heated debate [3,4], it is now widely accepted that the innexin gene family encodes invertebrate gap junction proteins [5,6]. Innexins are predicted to have four transmembrane helices like connexins, despite their lack of significant sequence similarity. At present, it is unclear whether the genetic relationship between connexin and innexin is a convergence or divergence [7,8]. Why different proteins whose sequences appear to be unlike are required for gap junction channels in nature is an intriguing question. This review focuses on the structural aspects of the two gap junction families to discuss the similarities and differences in gap junction channels. Technical aspects of structural studies by cryo-electron microscopy (EM) are also discussed.

## Crystallographic studies of connexin gap junction channels

Historically, chordate connexin gap junction channel structures were studied using electron crystallography as a two-dimensional (2D) array of channels is generated in native tissues or by recombinant expression systems [9–13]. All structures exhibit dodecameric subunits in a single full gap junction channel, some of which implicated models of channel closure, subunit rotation, and physical blockage with a plug [10,12,14]. The limited resolution of these structures, however, hampered elucidation of the structural details in terms of the helical arrangement of the transmembrane and cytoplasmic domains, and extracellular gap regions. The first high-resolution structure of Cx26 provided insight into these properties [15]. This structure allowed for the assignment of transmembrane helices (TM1–TM4), and visualized the N-terminus in the pore vestibule forming a funnel, leading to the interpretation that the channel was in an open state. Because of disorder due to artificial interactions caused by crystal contact, most of the cytoplasmic loop and C-terminal domain were not visible. Therefore, the gating mechanism induced by pH and chemicals thought to be associated with the cytoplasmic domains of connexin [16,17] remains to be determined. Alternative X-ray structures of Cx26 in crystallization buffer with and without calcium ions were recently reported [18]. This work claimed that the calcium ion-binding sites are located in the pore pathway around the TM1/first extracellular loop (E1) border that is closer to the extracellular gap region, whereby the electrostatic barrier changes, resulting in the exclusion of ions. Molecular dynamics simulation studies have been applied to the atomic model of Cx26, suggesting refined interpretations about the gating mechanism [19,20].

## Three-dimensional structure of INX-6ΔN by electron crystallography

**Fig. 1.**
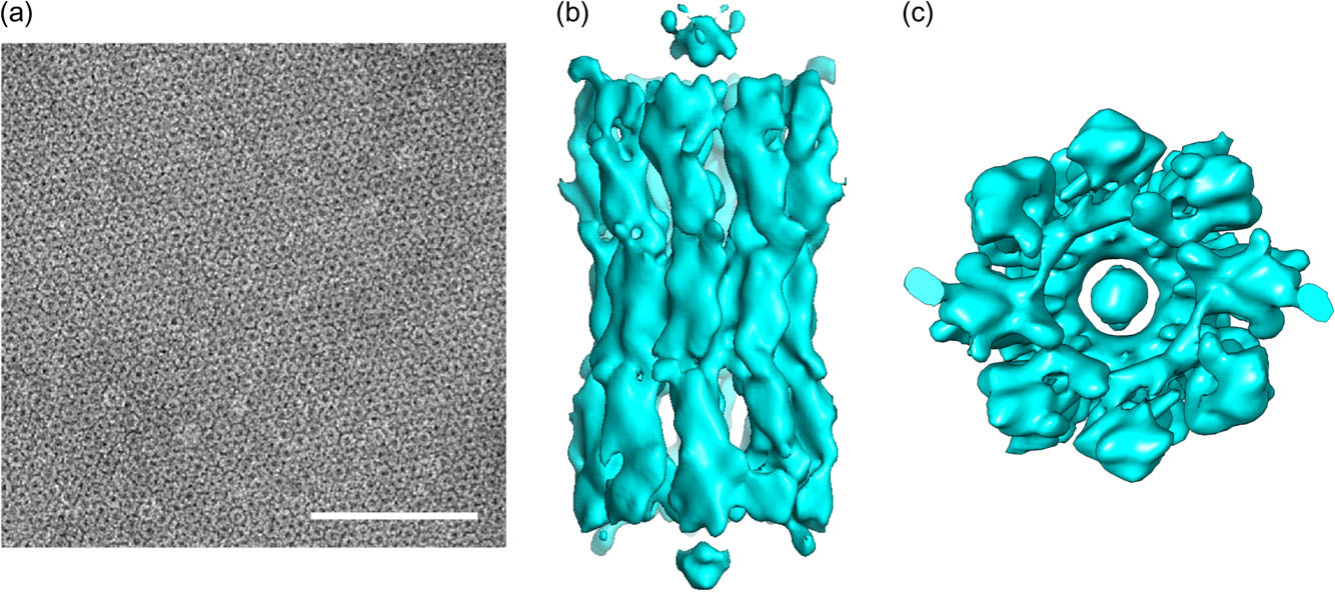
Three-dimensional structure of INX-6ΔN based on electron crystallography [22]. (a) Negatively stained electron micrograph of an INX-6ΔN 2D-crystal sheet. Bar = 100 nm. (b and c) Three-dimensional maps of INX-6ΔN at 10 Å resolution (b) side view and (c) top view.

No high-resolution innexin channel structure has yet been reported. Since the successful expression and purification of *C. elegans* innexin-6 (INX-6) [21], we have focused on structural studies of INX-6 gap junction channels using EM. First, purified INX-6 channels were crystallized in a 2D lipid bilayer. Wild-type channels were unfortunately not crystallized, but rather N-terminal deleted INX-6 (INX-6ΔN), in which 18 residues at the N-terminus were excluded, formed 2D crystals (Fig. 1a). The three-dimensional (3D) structure of INX-6ΔN was reconstructed at 10 Å resolution by electron crystallography (Fig. 1b and c) [22]. This structure revealed that the oligomeric component of a full gap junction channel is 16 subunits, unlike dodecameric connexin gap junction channels. As expected, the channel dimensions of INX-6ΔN were larger than those of Cx26. The comparison of structural and functional properties of connexin and innexin families has comprehensively described in the recent review by Skerrett and Williams [23]. The structure of INX-6ΔN exhibited four bulb densities in the channel pore pathway, but the interpretation was complicated due to the limited resolution and defective function caused by the N-terminal deletion [22].

## GraDeR for single particle cryo-EM

**Fig. 2.**
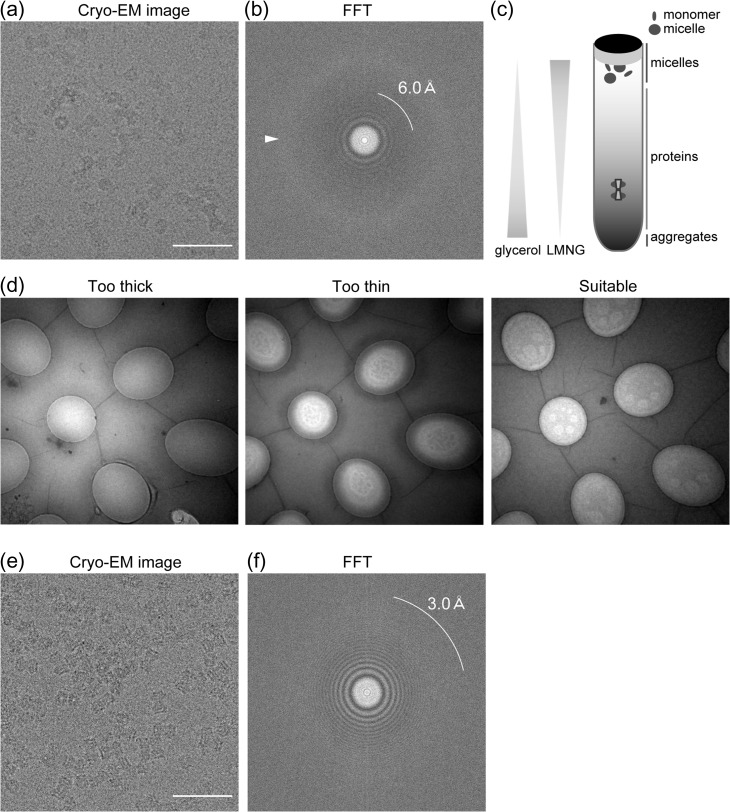
Cryo-EM data collection of wild-type INX-6 gap junction channels (modified from [24]). (a) Cryo-electron micrograph of INX-6 channels in thick ice containing 0.1% OGNG. Bar = 50 nm. (b) Fast-Fourier Transform image of (a). 6.0 Å resolution is indicated by the white line. Diffuse ice ring outside 6 Å resolution is visible (arrowhead), suggesting thick ice. (c) Schematic representation of GraDeR [35]. It should be noted that the glycerol concentration increases towards the bottom, and LMNG forms a reverse gradient in the ultracentrifuge tube. Free detergent micelles and monomers are removed by glycerol gradient centrifugation as only oligomeric INX-6 gap junction channels sink. (d) Search mode images of cryo-EM grids using purified INX-6 channels after GraDeR. Most holes have thick ice where only ambiguous particle images can be recorded (left). No particles are observed in excessively thin ice (middle), and suitably thin ice shows the contrast between the small white circles (right), and high-contrast particle images can be obtained from the grey area in these holes. (e) Cryo-electron micrograph of INX-6 channels after GraDeR showing side and top views. Bar = 50 nm. (f) Fast-Fourier Transform image of (e). 3.0 Å resolution is indicated by the white line. No ice ring is visible in this image.

To improve the resolution and elucidate the wild-type structure of INX-6, we performed single particle cryo-EM using the wild-type INX-6 proteins [24]. Optimal sample preparation for cryo-EM is essential for recording high-resolution electron micrographs. A suitably thin ice layer is typically transparent, allowing for detailed visualization of the recorded particles [25]. For INX-6, gel filtration elution containing 0.1% octyl glucose neopentyl glycol (OGNG) was first used to prepare the cryo-EM grid. As in previous studies [26–28], gel filtration peak fractions were concentrated as much as for 3D crystallization. With concentrated INX-6 proteins at 5–10 mg/ml, cryo-EM images showed visible particles of INX-6 with a good distribution and wide orientation (Fig. 2a). Thon rings from those images, however, did not extend beyond 6 Å resolution, but instead, the ice ring was visible (Fig. 2b), suggesting that the ice thickness of the grid was not optimized (i.e. too thick) [29]. Detergent micelles are factors that contribute to decrease the success ratio of good cryo-EM grids so that particles are contained within the thin ice layer [25,30]. There are a couple of ways to exclude detergent micelles without deteriorating membrane proteins, which have been applied for high resolution cryo-EM. One is exchanging the surrounding detergent micelles with amphipol [31–33]. Amphipol is not suitable for INX-6 channels, however, as they are denatured after gel filtration when using detergent-free buffer. Alternatively, reconstitution of membrane proteins into nano discs allowed for successful high-resolution cryo-EM analysis, such as for a TRPV channel [34]. Here, we used a recently developed method, GraDeR, which has good compatibility with INX-6 proteins [35].

GraDeR uses glycerol gradient centrifugation, which achieves mild conditions so that fragile membrane proteins are not denatured by the removal of free detergent micelles (Fig. 2c) [35]. In the ultracentrifuge tube for GraDeR, the concentration of glycerol increases towards the bottom whereas there is a reverse gradient of lauryl maltose neopentyl glycol (LMNG). This method is highly suitable for INX-6 channels [35]. Because the docked junction form of INX-6 is favoured for cryo-EM analysis, the original GraDeR protocol was slightly modified. For example, we used 500 mM NaCl instead of 150 mM, and a 5–25% glycerol gradient instead of a 5–30% gradient, with an ultracentrifuge tube for the Beckman SW41Ti rotor. The gel filtration eluate of INX-6 in 0.1% OGNG buffer was directly loaded onto the top of the gradient solution. An essential step was to add LMNG to the INX-6 eluate at 0.02% in advance; otherwise, channels were denatured after ultracentrifugation, suggesting that LMNG in the gradient buffer itself does not always ensure membrane protein stability. Due to the small number of results so far, it is unclear whether LMNG is generally applicable to any membrane proteins. Optimization of the centrifuge time, centrifuge force and other above-mentioned parameters is important, and depends on the membrane proteins to be studied.

## Selection of holes in the cryo-EM grid for high-quality images

When preparing cryo-EM grids with the VitrobotIV (FEI, Hillsboro, OR, USA), parameters such as the blotting time, blotting force, temperature and humidity are not very meaningful because the success ratio of the best cryo-EM grids is often subject to the climate. The contrast in the holes under the search mode is informative to discriminate which hole should be recorded. Hole contrast with a concave lens appearance usually corresponds to thick or unevenly flat ice (Fig. 2d, left). The particles from these holes often appear ambiguous with poor Thon rings in fast-Fourier transform (FFT) (Fig. 2a and b), and do not reach high resolution. Holes with grey spots (Fig. 2d, middle) on a bright background no longer contain particles, and comprise empty ice. The optimized flat ice shows grey and transparent contrast, including small white circles (Fig. 2d, right). These white circles are actually not open holes, but contain very thin empty ice. The particles are located in the grey contrast area in this hole, allowing for very sharp cryo-EM images with high contrast of the mostly side view particles (Fig. 2e), for which the FFT image often shows Thon rings with over 3 Å resolution (Fig. 2 f). While we do not evaluate the absolute thickness of the ice, this type of thin ice is often broken after electron exposure. The best flat ice holes appear in one grid with low frequency, typically less than 10% of total meshes. Because the ice condition is determined at the time of the freezing process, the most time-consuming step is preparing reproducibly good ice. To increase the success ratio, the quality of a cryo-EM image is checked every time the image is recorded. Because the sample exchange of the JEM-3000SFF (JEOL) electron microscope [36], for which all steps are manually operated, can be completed in 10 min, a low-quality cryo-EM grid is immediately replaced with a new one, allowing us to optimize the freezing condition based on rapid feedback. We selected high-quality cryo-EM images based on the following criteria: particles were clearly seen with sharp contrast in a cryo-EM image, Thon rings extended to at least 5 Å resolution, no ice ring appeared in FFT (Fig. 2 f), and 2D class averages exhibited the properties of secondary structures, specifically transmembrane helices.

## Structure determination of the wild type INX-6 channel

**Fig. 3.**
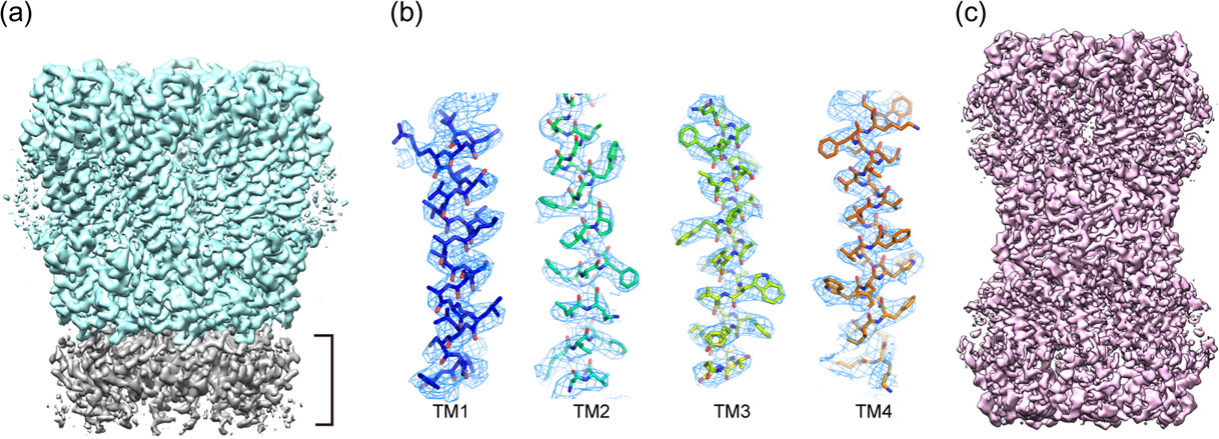
Cryo-EM structure of INX-6 gap junction channels (modified from [24]). (a) Density map of an INX-6 hemichannel at 3.3 Å resolution (masked). Grey density marked by the bracket corresponds to part of the opposite hemichannel. (b) Density maps of transmembrane helices of INX-6. Atomic models of TM1–TM4 are superimposed on the density map (blue mesh). (c) Density map of an INX-6 gap junction channel at 3.6 Å resolution (masked).

The particle box size of 160 × 160 pixels covering the INX-6 hemichannel was useful for auto particle picking to work (swarm function of EMAN2 [37]). After 3D classification of more than 340 000 hemichannel particles, one class reached 3.3 Å resolution (masked) with C8 symmetry, the map of which represents the side-chain densities (Fig. 3a and b), and allowed us to generate a *de novo* model as the first high-resolution structure in the innexin family. For the map calculation of a full gap junction channel, particles were boxed with a box size of 200 × 200 pixels. This was more complicated because the particle shape was not well suited for auto particle picking. Approximately 35 000 particles of a junction channel were collected, and the 3D refinement was successful including all the particles when D8 symmetry was imposed (Fig. 3c). The quality of the INX-6 gap junction channel map at 3.6 Å resolution (masked) was not as good as that of the hemichannel map, but the atomic model of a hemichannel could be superimposed on the gap junction map, and model refinement was successful.

**Fig. 4.**
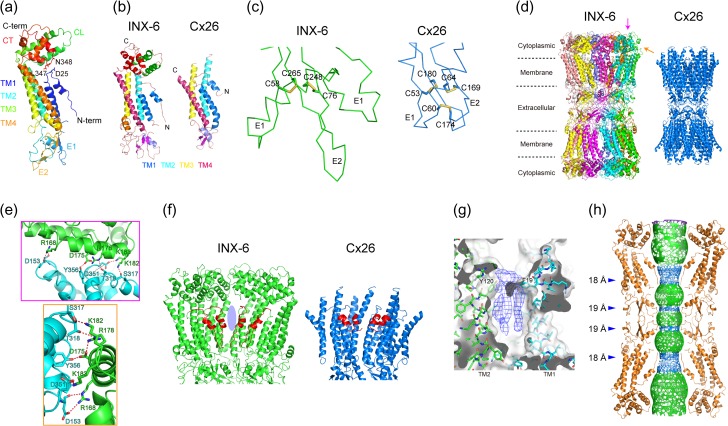
Atomic structure of INX-6 and similarity to Cx26 (modified from [24]). (a) Model of INX-6 monomer. D25, L347 and N348, represented by stick models, are within interacting distance. (b) Similar arrangement of INX-6 and Cx26 monomers. The secondary structures of the two structures are coloured the same. (c) Disulphide bonds (shown as stick model) formed in the extracellular loops of INX-6 and Cx26. (d) Gap junction structures of INX-6 and Cx26. Each subunit of INX-6 is represented by a different colour. The scales for these two structures are matched. The magenta and orange arrows indicate the view angles of panels in (e). (e) Polar interactions in the cytoplasmic domains between adjacent subunits. The models and residue labels are colour coded according to the subunits. The two panels are viewed from different angles. The outer box colour (magenta or orange) corresponds to the arrow colour in (d). (f) N-terminal funnel of INX-6 and Cx26. Only four subunits in a hemichannel are shown for clarity. The N-terminal portion is coloured in red. Blue oval shows space between adjacent two subunits of INX-6. (g) Density found in spaces between adjacent subunits marked with a blue oval in (f). (h) Pore pathway of an INX-6 gap junction channel shown as mesh representation. Narrow sites (blue mesh) are marked by arrowheads, with diameters of 18 Å or 19 Å.

The monomeric structure of INX-6 has a sea horse-like appearance when the cytoplasmic domain is placed up (Fig. 4a). The innermost helix is TM1, facing the pore pathway, with the N-terminal portion having a short hairpin loop and the N-terminal helix located in the pore vestibule. The cytoplasmic domains of INX-6, which are not visible in the Cx26 structure [15], form a core comprised of a cytoplasmic loop and C-terminal domain, and have a kinked connection to TM2–TM4. The two residues of Leu347 and Asn348 at the C-terminus are able to contact Asp25 in the N-terminal loop, whereby the conformational change in the cytoplasmic domains can be conveyed to the N-terminus (Fig. 4a). Pro122 in TM2 is strictly conserved in the innexin family, contributing to the kinking of TM2. E1 contains an α helix, and there is a β hairpin in the second extracellular loop (E2).

The arrangement of an INX-6 monomer is very similar to that of the Cx26 monomer determined by X-ray (Fig. 4b), while the cytoplasmic domains of Cx26 are partially disordered. Common properties are the N-terminal helix, the assignment of TM1–TM4, the proline kink in TM2, the short α helix in E1, and the β hairpin in E2. When the Dali server [38] was used to search for protein structures similar to the INX-6 monomeric structure, Cx26 (PDB code: 2zw3) was given a score of 7.9, suggesting that Cx26 and INX-6 have a highly similar monomeric structural organization. Connexins have three highly conserved cysteines in each extracellular loop, forming three disulphide bonds between E1 and E2 (Fig. 4c) [15]. Innexins have two conserved cysteines in E1 and E2, which appear to form disulphide bonds in the INX-6 structure as in Cx26. Interestingly, the special distribution of the two disulphide bonds in INX-6 well correspond to two of the three disulphide bonds in Cx26 (Fig. 4c), suggesting that these four extracellular cysteines are conserved between INX-6 and Cx26, which also implies a genetic correlation between the connexin and innexin families. The proline in TM2 that causes a helix kink is strongly conserved in both families, which also supports a genetic correlation between these two families.

## Structure of an INX-6 gap junction channel

As reported previously [22], a full gap junction channel of INX-6 comprises 16 subunits, consistent with the larger overall dimensions than those of Cx26 (Fig. 4d). The helix-rich cytoplasmic domains of INX-6 form a continuous roof in an octameric hemichannel and multiple polar interactions are generated between adjacent subunits in this domain (Fig. 4e). We termed this domain the ‘cytoplasmic dome’, as it has the appearance of a dome stadium. There is a possibility that connexins categorized in the alpha type subfamily have a similar cytoplasmic dome since their C-terminal portions are thought to comprise more than 50 amino acid residues. However, this may not apply to all connexins because some isoforms in other connexin subfamilies (beta, gamma, delta and epsilon [39]) would have a short C-terminus. For instance, Cx26 is a member of the beta type subfamily and has a C-terminal tail containing less than 20 amino acid residues. This would be insufficient to form the same cytoplasmic dome as INX-6. There are 32 α helices in the transmembrane domain, and the channel pore entrance harbours the N-terminal funnel, consistent with the Cx26 structure (Fig. 4f) [15]. The N-terminal funnel is considered to play an essential role in the activity of gap junction channels, whereas a different subunit number is acceptable between the two families. The structural property specific to INX-6 is the spacing between the adjacent transmembrane helix bundles. While nothing was observed in the space of the 2D crystallographic structure of INX-6ΔN (Fig. 2b) [22], the high-resolution map of INX-6 shows the unassigned densities (Fig. 4g). These may be lipid or detergent molecules, and they may play a role in stabilizing the transmembrane domains *in vivo* as a previous study suggested that the transmembrane helices of innexin channels have loose packing [40]. The extracellular E1 short α helices have a radial distribution, narrowing the pore diameter, which is surrounded by β hairpins in E2 to seal the pore pathway.

Whether the connexin and innexin families are genetically correlated remains unclear due to the lack of sequence similarity of these proteins [7,8]. Our results demonstrate that there are several common functional/structural properties between INX-6 and Cx26, and that the structural properties of the two families are highly correlated. Given that some key residues may be conserved, these two families are possibly derived from a common genetic ancestor followed by a long divergence time. Further studies are needed to clarify whether innexin and connexin can be categorized to a gap junction superfamily.

## Pore pathway of an INX-6 gap junction channel and activity regulation

The channel pore pathway of an INX-6 gap junction channel becomes narrow at four sites. Two of them are surrounded by the N-terminal funnel, and other two are at the E1 short α helices (Fig. 4 h). The pore diameter is smallest at the N-terminal funnel, but is still 18 Å, which allows for passage of a single α helix. Six residues at the N-terminus are not visible, making it difficult to interpret whether the conformation is open or closed. According to the density map, however, a straightforward interpretation is that the structure is in an open conformation. The helix-rich cytoplasmic dome is in close proximity with the N-terminal funnel, like a lid, from the cytoplasmic side, whereby any conformational changes taking place in the cytoplasmic dome are immediately transferred to the activity regulation by the N-terminal funnel. While the cytoplasmic domains of Cx26 are not completely resolved, low pH conditions and mutation or deletion at the connexin C-terminus reportedly modulate the gap junction channel activity [41–43]. It has been known that invertebrate gap junctions are also modulated by cytoplasmic pH [44–48]. Our INX-6 structure first revealed the cytoplasmic domains of gap junction channels and may provide clues into how gap junction channel activity is regulated by these domains. Unfortunately, a closed-state structure of INX-6 has not yet been determined, and thus the closing mechanism involving the cytoplasmic domains is a challenge for the future. Nevertheless, it should be noted that the N-terminal domain, forming a funnel in Cx26 and INX-6, is highly associated with gap junction channel activity, and the innexin and connexin families may share a common mechanism using the N-terminal funnel.

## Prospects

It is quite peculiar that proteins with unlike sequences can form gap junction channels in nature. The atomic structures of INX-6 and Cx26 reveal that while these two proteins differ in terms of subunit number, channel dimension and pore diameter, they share a similar monomeric structure, N-terminal funnel and tsuzumi-shaped gap junction channel structure. The biologic significance of the presence of larger gap junction channels in invertebrates than in vertebrates remains to be elucidated. Furthermore, the structure of INX-6 appears open like the Cx26 X-ray structure [15], and therefore it is difficult to understand how the channel closes. To address this question, it is necessary to elucidate more structures of gap junction channels, specifically in a closed state. Because gap junction channels form a large oligomeric complex containing more than 10 subunits, single particle cryo-EM would be suitable for high-resolution structural studies and would be a key strategy for elucidating the gating mechanism of gap junction channels.

## Abbreviations

Cx, connexin; INX, innexin; NT, amino terminal domain; CT, carboxyl terminal domain; CL, cytoplasmic loop; E1, first extracellular loop; E2, second extracellular loop; TM, transmembrane helix; EM, electron microscopy; OGNG, octyl glucose neopentyl glycol; LMNG, lauryl maltose neopentyl glycol.
